# Sensor Signal and Information Processing III

**DOI:** 10.3390/s20236749

**Published:** 2020-11-26

**Authors:** Wai Lok Woo, Bin Gao

**Affiliations:** 1 Department of Computer and Information Sciences, Northumbria University, Newcastle upon Tyne NE1 8ST, UK; 2 School of Automation Engineering, University of Electronic Science and Technology of China, Chengdu 611731, China; bin_gao@uestc.edu.cn

Sensors are one of the key factors in the success of the Internet of Things (IoT); however, these sensors are not conventional types that simply convert physical variables into electrical signals. They have substantially evolved into something more complex and sophisticated and perform a technically and economically viable role in most IoT applications. Sensors have traditionally been functionally simple devices that convert physical variables into electrical signals or create changes in electrical properties. While this functionality is a crucial starting point, sensors have extra functionalities such as very low power consumption, self-identification and self-verification, and they are wireless and physically small enough to “disappear” unobtrusively into any environment. In addition, information from multiple sensors can be combined and correlated to make conclusions about latent problems, for example, temperature sensor and vibration sensor data can be used to detect the onset of mechanical failure. In some cases, multiple sensor functions are available in one device, in others, the functions are combined in software to create a “soft” sensor. It has become evident that sensor intelligence, apart from facilitating connectivity, also has many other benefits including predictive maintenance, more flexible manufacturing, and improved productivity.

This is the third in a series of Special Issues dedicated to Sensor Signal and Information Processing (SSIP) [1]. The first two series of SSIP were published in 2018 and 2019, respectively [2,3,4]. SSIP has become an overarching field of research that is focused on the mathematical foundations and practical applications of signal processing algorithms that learn, reason, and act. It bridges the boundary between theory and application to develop novel, theoretically-inspired methodologies that target both longstanding and emergent signal processing applications. The core of SSIP lies in its use of nonlinear and non-Gaussian signal processing methodologies combined with convex and nonconvex optimization. SSIP encompasses new theoretical frameworks for statistical signal processing (e.g., deep learning, latent component analysis, tensor factorization, and Bayesian methods) coupled with information theoretical learning, and novel developments in these areas are specialized in the processing of a variety of signal modalities including audio, bio-signals, multi-physics signals, images, multispectral and video, among others. In recent years, many signal processing algorithms have incorporated some forms of computational intelligence as part of their core framework in problem solving. These algorithms have the capacity to generalize and discover knowledge for themselves and to learn new information whenever unseen data are captured. The focus of this Special Issue is on a broad range of sensors, signal and information processing involving the introduction and development of new advanced theoretical and practical algorithms. It includes twenty works focused on sensor signal and information processing based on diverse technologies for different applications.

Permutation entropy (PE) is a powerful complexity measure for analyzing time series and it has the advantage of easy implementation and high efficiency. In order to improve the performance of PE, Li et al. [5] have recently proposed improved methods by introducing amplitude information and distance information. Weighted-permutation entropy (W-PE) uses variance information to weight each arrangement pattern, which has good robustness and stability in the case of high noise level and can extract complexity information from data with spike features or abrupt amplitude changes. Dispersion entropy (DE) introduces amplitude information by using the normal cumulative distribution function. It is not only able to detect the change of simultaneous frequency and amplitude, but also is superior to the PE method in distinguishing different data sets. Reverse permutation entropy (RPE) is defined as the distance to white noise in the opposite trend to PE and W-PE, and it has high stability for time series with varying lengths. To further improve the performance of PE, the authors have proposed a new complexity measure known as reverse dispersion entropy (RDE), which takes PE as its theoretical basis and combines the advantages of DE and RPE by introducing amplitude information and distance information.

Along the same line of time series research, Parathai et al. [6] have proposed a solution based on complex non-negative matrix factorization (CMF) for events classification from a noisy mixture. It encodes the spectra pattern and estimates the phase of the original signals in the time-frequency representation through an adaptive *L*_1_ sparsity CMF algorithm. The features enhance the efficiency of the temporal decomposition process. The support vector machine-based one-versus-one strategy was applied with a mean supervector to categorize the demixed sound into the matching sound-event class. The rapid development of sensor technology has given rise to the emergence of huge amounts of tensor data. For various reasons, such as sensor failures and communication loss, the tensor data may be corrupted by both small noises and gross corruptions. Fang et al. [7] have studied the Stable Tensor Principal Component Pursuit (STPCP), which aims to recover a tensor from its corrupted observations. The proposed model is based on the tubal nuclear norm, which has shown superior performance in comparison with other tensor nuclear norms. It is shown theoretically that the underlying tensor and the sparse corruption tensor can be stably recovered under tensor incoherence conditions.

Indoor positioning using Wi-Fi signals is an economic technique. Its main drawback is that multipath propagation distorts these signals, thus leading to inaccurate localization. One approach to improve the positioning accuracy consists of using fingerprints based on channel state information (CSI). Wang et al. [8] have proposed a new positioning method that consists of three stages. In the first stage, a model is built for the fingerprints of the environment. This model obtains a precise interpolation of fingerprints at positions where a fingerprint measurement is not available. In the second stage, the model is then used to obtain a preliminary position estimate based only on the fingerprint measured at the receiver’s location. Finally, the preliminary estimation with the dynamical model of the receiver’s motion are used to obtain the final estimation. The obtained experimental results from the proposed method show that it has a promising future.

The automatic sleep stage classification technique can facilitate the diagnosis of sleep disorders and free medical experts from labor-consuming work. Shen et al. [9] have proposed an improved model-based essence features that combines locality energy and dual state space models for automatic sleep stage detection on single-channel electroencephalograph signals. The experimental results have shown high classification accuracy compared with state-of-the-art methods. Similarly, motor imagery (MI)-based brain-computer interface (BCI) systems detect electrical brain activity patterns through electroencephalogram signals to forecast user intention while performing movement imagination tasks. As the microscopic details of individuals’ brains are directly shaped by their rich experiences, musicians can develop certain neurological characteristics, such as improved brain plasticity following extensive musical training. Riquelme-Ros et al. [10] have developed a new approach to assess the performance of pianists as they interacted with an MI-based BCI system and compared it with that of a control group. The outcome indicates that musical training could enhance the performance of individuals using BCI systems.

In image processing, the recognition of scene changes plays an essential role in a variety of real-world applications, such as scene anomaly detection. Most scene understanding research has focused on static scenes and most existing scene change captioning methods detect scene changes from single-view color images, neglecting the underlying three-dimensional structures. Previous three-dimensional scene change captioning methods have used simulated scenes consisting of geometry primitives, making them unsuitable for real-world applications. To solve these problems, Qiu et al. [11] have proposed an end-to-end framework for describing scene changes from various input modalities, namely, RGB images, depth images, and point cloud data, which are available in most robot applications. In a similar development, Kim and Kudo [12] have proposed a new class of nonlocal Total Variation (TV), in which the first derivative and the second derivative are mixed. Since most existing TV only considers the first-order derivative, it suffers from problems such as staircase artifacts and loss in smooth intensity changes for textures and low-contrast objects, which is a major limitation in improving image quality. The proposed nonlocal TV combines the first and second order derivatives to effectively preserve smooth intensity changes. The non-rigid multi-modal 3D medical image registration is highly challenging due to the difficulty in constructing a similarity measure and the solution of non-rigid transformation parameters. Yang et al. [13] have proposed a novel structural representation-based registration method to address these problems. Firstly, an improved modality independent neighborhood descriptor (MIND) based on the foveated nonlocal self-similarity is designed for effective structural representations. Subsequently, the foveated MIND-based spatial constraint is introduced into the Markov random field (MRF) optimization to reduce the number of transformation parameters and restrict the calculation of the energy function in the image region involving non-rigid deformation. Finally, the accurate and efficient 3D medical image registration is realized by minimizing the similarity measure-based MRF energy function. Experiments on real magnetic resonance and ultrasound images with unknown deformation were also done to demonstrate the practicality and superiority of the method.

Deformable image registration is still a challenge when the considered images have strong variations in appearance and large initial misalignment. A huge performance gap currently remains for fast-moving regions in videos or strong deformations of natural objects. Ha et al. [14] have combined a U-Net architecture that is weakly supervised with segmentation information to extract semantically meaningful features with multiple stages of non-rigid spatial transformer networks parameterized with low-dimensional B-spline deformations. The models are compact, very fast in inference, and demonstrate clear potential for a variety of challenging tracking and/or alignment tasks in computer vision and medical image analysis. Compressive sensing (CS) spectroscopy is well known for developing a compact spectrometer that consists of two parts: compressively measuring an input spectrum and recovering the spectrum using reconstruction techniques. Kim et al. [15] have proposed a residual convolutional neural network for reconstructing the spectrum from the compressed measurements. The proposed network comprises learnable layers and a residual connection between the input and the output of these learnable layers. The proposed network has produced stable reconstructions under noisy conditions.

Although deep learning has achieved great success in many applications, its usage in communication systems has not been well explored. Zha et al. [16] investigated algorithms for multi-signal detection and modulation classification, which are significant in many communication systems. In their work, a deep learning framework for multi-signal detection and modulation recognition is proposed. Compared to some existing methods, the signal modulation format, center frequency, and start-stop time can be obtained from the proposed deep learning scheme. The control effect of various intelligent terminals is affected by the data sensing precision. Usually, the filtering method is based on the typical soft computing method used to promote the sensing level. Bai et al. [17] have proposed a neuron-based Kalman filter to overcome limitations due to the difficult recognition of the practical system and the empirical parameter estimation in the traditional Kalman filter. The neuro units optimize the filtering process to reduce the effect of the unpractical system model and hypothetical parameters. It is shown that the neuro-filter is effective in noise elimination within the soft computing solution. IoT systems generate a large volume of data all the time. How to choose and transfer which data are essential for decision-making is a challenge. This is especially important for low-cost and low-power designs where data volume and frequency are constrained by the protocols. Tsapparellas et al. [18] have presented an unsupervised learning approach using Laplacian scores to discover which types of sensors can be reduced without compromising the decision-making. Here, a type of sensor is a feature. A comparative study has shown that when fewer types of sensors are used; the accuracy of the decision-making remains at a satisfactory level.

In defect detection, the surface quality of aluminum ingot is crucial for subsequent products, so it is necessary to adaptively detect different types of defects on the surface of milled aluminum ingots. Liang et al. [19] have proposed a two-stage detection to quickly apply the calculations to a real production line. A mask gradient response-based threshold segmentation is developed to extract the target defects. An inception-v3 network with a data augmentation technology and the focal loss is further proposed to overcome the class imbalance problem and improve the classification accuracy. The gearbox is one of the most fragile parts of a wind turbine. Yin et al. [20] have developed a fault diagnosis method for wind turbine gearboxes based on optimized long short-term memory neural networks with cosine loss (Cos-LSTM). The loss is converted from Euclid space to angular space by cosine loss, thus eliminating the effect of signal strength and improving the diagnosis accuracy. The energy sequence features and the wavelet energy entropy of the vibration signals are used to evaluate the Cos-LSTM networks. The effectiveness of the method is verified with the fault vibration data collected on a gearbox fault diagnosis experimental platform. To suppress noise in signals, a denoising method on the basis of the singular value decomposition (SVD) and the Akaike information criterion (AIC) is proposed in [21], which is called AIC-SVD. To verify the effectiveness of AIC-SVD, it is compared with wavelet threshold denoising and empirical mode decomposition with a Savitzky–Golay filter. The proposed method is self-adaptable and robust while avoiding the occurrence of over-denoising.

In wireless communication, a frequency-hopping (FH)-based dual-function multiple-input multiple-output (MIMO) radar communications system enables simultaneous implementation of a primary radar operation and a secondary communication function. The set of transmit waveforms employed to perform the MIMO radar task is generated using FH codes. However, as the radar channel is time-variant, it is necessary for a successive waveform optimization scheme to continually obtain target feature information. Yao et al. [22] have developed a method to enhance target detection and feature estimation performance by maximizing the mutual information (MI) between the target response and the target returns, and then minimizing the MI between successive target-scattering signals. Chen et al. [23] propose a geometric calibration method using sparse recovery to remove the linear array push-broom sensor bias. By using the sparse recovery method, the number and distribution of ground control points needed are greatly reduced. Meanwhile, the proposed method effectively removes short-period errors by recognizing periodic wavy patterns in the first step of the process. Finally, Sun et al. [24] have proposed a blind modulation classification method based on compressed sensing using a high-order cumulant and cyclic spectrum combined with the decision tree-support vector machine classifier. The proposed method solves the problem of low identification accuracy under single-feature parameters and reduces the performance requirements of the sampling system. Through calculating the fourth-order, eighth-order cumulant and cyclic spectrum feature parameters by breaking through the traditional Nyquist sampling law in the compressed sensing framework, six different cognitive radio signals are effectively classified. The results indicate that accurate and effective modulation classification can be achieved, and this provides a theoretical basis and technical advance for the field of optical-fiber signal detection.
